# A metabonomic study of cardioprotection of ginsenosides, schizandrin, and ophiopogonin D against acute myocardial infarction in rats

**DOI:** 10.1186/1472-6882-14-350

**Published:** 2014-09-23

**Authors:** Miaomiao Jiang, Liyuan Kang, Yi Wang, Xiaoping Zhao, Xuan Liu, Lei Xu, Zheng Li

**Affiliations:** Tianjin State Key Laboratory of Modern Chinese Medicine, Tianjin University of Traditional Chinese Medicine, 88 Yuquan Road, Nankai District, Tianjin, 300193 China; Pharmaceutical Informatics Institute, College of Pharmaceutical Sciences, Zhejiang University, 866 Yuhangtang Road, Xihu District, Hangzhou 310058 China; College of Preclinical Medicine, Zhejiang Chinese Medical University, 548 Binwen Road, Binjiang District, Hangzhou, 310053 China

**Keywords:** Metabonomics, Combined medications, ^1^H NMR, Ginsenosides, Schizandrin, Ophiopogonin D, Acute myocardial infarction

## Abstract

**Background:**

Metabonomics is a useful tool for studying mechanisms of drug treatment using systematic metabolite profiles. Ginsenosides Rg1 and Rb1, ophiopogonin D, and schizandrin are the main bioactive components of a traditional Chinese formula (Sheng-Mai San) widely used for the treatment of coronary heart disease. It remains unknown the effect of individual bioactive component and how the multi-components in combination affect the treating acute myocardial infarction (AMI).

**Methods:**

Rats were divided into 7 groups and dosed consecutively for 7 days with mono and combined-therapy administrations. Serum samples were analyzed by proton nuclear magnetic resonance (^1^H NMR) spectroscopy. Partial least squares discriminate analysis (PLS-DA) was employed to distinguish the metabolic profile of rats in different groups and identify potential biomarkers.

**Results:**

Score plots of PLS-DA exhibited that combined-therapy groups were significantly different from AMI group, whereas no differences were observed for mono-therapy groups. We found that AMI caused comprehensive metabolic changes involving stimulation of glycolysis, suppression of fatty acid oxidation, together with disturbed metabolism of arachidonic acid, linoleate, leukotriene, glycerophospholipid, phosphatidylinositol phosphate, and some amino acids. *β*-hydroxybutyrate, cholines and glucose were regulated by mono-therapy of schizandrin and ginsenosides respectively. Besides these metabolites, combined-therapy ameliorated more of the AMI-induced metabolic changes including glycerol, and *O*-acetyl glycoprotein. A remarkable reduction of lactate suggested the therapeutic effect of combined-therapy through improving myocardial energy metabolism.

**Conclusions:**

This study provided novel metabonomic insights on the mechanism of synergistic cardioprotection of combined-therapy with ginsenosides, schizandrin, and ophiopogonin D, and demonstrated the potential of discovering new drugs by combining bioactive components from traditional Chinese formula.

**Electronic supplementary material:**

The online version of this article (doi:10.1186/1472-6882-14-350) contains supplementary material, which is available to authorized users.

## Background

Myocardial infarction (MI), as a common presentation of coronary artery disease, has become a leading cause of mortality and morbidity in both developed and developing countries. Acute myocardial infarction (AMI) is a type of MI with ST-elevation due to myocardial cell death caused by thrombotic occlusion of the coronary artery [1, 2]. In cardiovascular diseases such as AMI, the heart undergoes a “metabolic shift” with large scale perturbations in metabolism during the pathological progression [3]. AMI has been recognized to cause dysfunction of energy metabolism, oxidative stress and inflammation in plasma [4, 5]. Metabonomic profiling, with a particular focus on monitoring the perturbations in the concentrations of endogenous low molecular weight metabolites, provides a discovery tool for the pharmacological discipline of cardiovascular disease and medications [6].

Sheng-Mai San (SMS), a traditional Chinese formula widely used for the treatment of coronary heart disease, consists of three herbal materials of *Radix ginseng*, *Radix ophiopogonis* and *Fructus schisandrae*. Ginsenoside Rg1 [7], ginsenoside Rb1 [8], ophiopogonin D [9], and schizandrin [10] from each composition material have been suggested to be the main active components of SMS responsible for cardioprotection [11]. However, it remains unknown the mono-therapeutic effect of individual bioactive component and how the multi-components in combination regulate the metabolic profiles of AMI rats *in vivo*.

In this study, serum metabolic profiling based on proton nuclear magnetic resonance (^1^H NMR) spectroscopy was performed to discover the metabolite modulation in AMI rats treated with mono-therapy and combined-therapy of ginsenoside Rg1, ginsenoside Rb1, ophiopogonin D, and schizandrin respectively. We aimed to compare the regulatory effects of the combination with its 4 individual constituents to discover potential biomarkers that could predict the cardioprotection of the combined-therapy by using multivariate data analysis.

## Methods

### Chemicals

Analytical grade chloral hydrate, sodium chloride, sodium dihydrogen phosphate (NaH_2_PO_4_) and disodium hydrogen phosphate (Na_2_HPO_4_) were obtained from Sigma-Aldrich Chemical Co. (St. Louis, MO, USA). Deuterium oxide (D_2_O, 99.9% in D) was bought from Cambridge Isotope Laboratories, Inc. (MA, USA). Ginsenoside Rg1, ginsenoside Rb1, ophiopogonin D, and schizandrin (purity > 98%) were purchased from Shanghai Win herb Medical Technology Co., Ltd (Shanghai, P.R. China).

### Animal experiments and sample collection

Sixty-four adult male Sprague–Dawley rats (250–260 g) were purchased from Beijing HFK Bioscience Co. Ltd. (Beijing, China) and maintained under standard conditions with free access to rat diet and distilled water during the experimental period. Animals used in this study were maintained in accordance with internationally accepted guidelines for use of animals and the procedure was approved by the College of Animal Ethics Committee, Tianjin University of Traditional Chinese Medicine (TCM-LAEC2014001).

The acute myocardial infarction (AMI) model was carried out by left anterior descending coronary artery ligation (LADCA) as previously reported [12]. All the rats were fixed on pad in a position on the back, and the chest was opened by a middle thoracotomy in the fourth left intercostal space under sterile conditions. After pericardiotomy, the heart was rapidly exteriorized and a 6–0 black silk ligature was securely ligated under the LADCA to form an occlusion. To ascertain that the AMI model had been successful, electrocardiograms were recorded after ligation for 15 minute using a Biopac MP-100 data acquisition system (BIOPAC Systems, Inc. CA, USA) to obtain an elevation of ST-segment (more than 0.2 mV). The sham group was operated upon using the same process as previously mentioned, except for the ligation step. In total, 37 AMI rats and 6 sham rats survived.

The AMI rats were randomly divided into six groups (n ≥ 4) and dosed once daily consecutively for 7 days according to different administrations, including AMI model group (AMI), ginsenoside Rg1 and ginsenoside Rb1 treatment group (GB), ophiopogonin D treatment group (OD), schizandrin treatment group (SC), ginsenosides combined with schizandrin treatment group (SGB), ginsenosides combined with schizandrin and ophiopogonin D treatment group (SGBO). Since Radix ginseng was the monarch of SMS prescription and ginsenosides were considered to be the main active constituents of SMS, we did not design the treatment group of schizandrin combined with ophiopogonin D. Furthermore, ginsenosides Rg1 and Rb1, together with ophiopogonin D were all saponin compounds, so we also did not consider the treatment group of ginsenosides Rg1 and Rb1 combined with ophiopogonin D. During the experimental procedures, another 6 rats were sacrificed. Ginsenoside Rg1 (40 mg/kg/day), ginsenoside Rb1 (40 mg/kg/day) and schizandrin (4 mg/kg/day) were administered intravenously, ophiopogonin D was administered intragastricly at a dose of 1 mg/kg/day.

Echocardiography was performed with VeVo2100 Imaging System (Visual sonics Inc., Toronto, Canada).The left ventricular (LV) ejection fraction (EF) and fractional shortening (FS) were taken as measurements of LV systolic function [13]. Blood samples were collected at day 7 and centrifuged (4000 rpm) for 10 min to obtain serum. Serum samples were snap-frozen in liquid nitrogen immediately and stored at −80°C in a freezer for NMR analysis.

### Sample preparation and NMR spectroscopy

Each serum sample (300 μL) was mixed with 250 μL of saline solution (containing 50% D_2_O, 0.9% NaCl) and transferred into 5 mm NMR tubes for NMR analysis. All NMR spectra of serum were recorded on a Bruker AVIII HD 600 MHz NMR spectrometer (600.25 MHz for proton frequency) with a 5 mm BBO H&F cryoprobe. One-dimensional ^1^H NMR spectra were acquired at 298 K using the first increment of the gradient selected NOESY pulse sequence (NOESYGPPR1D) with water presaturation during both relaxation delay (2 s) and mixing time (80 ms). The 90° pulse length was adjusted to about 11 μs for each sample. A total of 16 transients were collected into 64 k data points with spectral width of 18 kHz. All free induction decays were zero-filled to 128 k and multiplied by an exponential function with a line broadening 0.5 Hz prior to Fourier transformation.

For signal assignment, a series of two-dimensional NMR (2D NMR) spectra were acquired for selected samples including ^1^H, ^1^H-correlation spectroscopy (COSY), ^1^H, ^1^H-total correlation spectroscopy (TOCSY), *J*-resolved Spectroscopy (*J*-res), ^1^H, ^13^C- heteronuclear single quantum correlation (HSQC) and heteronuclear multiple bond correlation spectroscopy (HMBC).

### NMR spectra process and analysis

All ^1^H NMR spectra were manually corrected for phase and baseline distortions within MestReNova 5.3.1 (Mestrelab Research S. L., Spain), and the chemical shifts were referenced to the methyl proton signal of alanine at *δ* 1.54. The spectral regions of *δ* 0.5-9.0 were integrated into bins of 0.004 ppm. Regions at *δ* 4.35-6.23 were discarded to eliminate the effects of imperfect water saturation. All remaining1653 segments in *δ* 0.5-4.34 and *δ* 6.24-9.0 were then normalized to the total integrated area of spectra, and then mean-centered and divided by the square root of standard deviation of each variable (pareto-scaling). Multivariate data analysis was conducted for the centered and scaling data with MetaboAnalyst 2.0 (http://ww.metaboanalyst.ca/) [14]. Principal component analysis (PCA) was performed to check outliers in the data set. Partial least squares discriminate analysis (PLS-DA) was carried out to identify metabolites significantly contributing to the group differentiation. The NMR data was used as X-matrix with log-transformation and pareto-scaling, and group information was used as Y-matrix. Model quality was assessed with R^2^ indicating the validity of models against over fitting and Q^2^ representing the predictive ability. Potential variables of interest were identified based on the loading scores and variable influence on projection (VIP). The statistical significance of these variables was calculated by t-test (*p* < 0.05).

## Results and discussion

### Echocardiographic assessment

We assessed the systolic function of rats from different groups using echocardiography. Summary data for the ejection fraction and fractional shortening was shown in Table 1. A trend of improvement by the combination-therapy e.g. SGBO can be observed, however it did not reach statistical significant level.

**Table 1:** **Summary of echocardiographic data**

Treatment	n	LVEDV (μL)	LVESV (μL)	LVIDd (mm)	LVIDs (mm)	EF (%)	FS (%)
Sham	5	86.56 ± 44.57	18.80 ± 15.31	4.27 ± 0.94	2.19 ± 0.76	78.05 ± 13.82	48.52 ± 14.48
AMI	6	188.25 ± 37.24	66.70 ± 30.09	6.10 ± 0.53	3.84 ± 0.76	64.04 ± 17.82	36.82 ± 12.60
GB	5	175.05 ± 43.22	67.49 ± 61.13	5.89 ± 0.65	3.67 ± 1.37	65.56 ± 23.72	38.95 ± 16.53
SC	5	203.90 ± 142.63	61.60 ± 129.67	6.12 ± 1.70	3.83 ± 2.32	67.75 ± 23.51	41.20 ± 18.31
OD	6	169.89 ± 35.39	43.16 ± 18.77	5.83 ± 0.52	3.21 ± 0.61	75.39 ± 7.08	45.30 ± 6.83
SGB	5	154.82 ± 35.56	57.41 ± 32.84	5.59 ± 0.56	3.58 ± 0.80	64.79 ± 12.06	36.43 ± 8.23
SGBO	4	123.89 ± 45.16	35.87 ± 28.88	5.04 ± 0.81	2.87 ± 0.91	72.96 ± 14.07	43.58 ± 11.91

LVEDV, left ventricular end-diastolic volume; LVESV, left ventricular end-systolic volume; LVIDd, left ventricular internal diameter in diastole; LVIDs, left ventricular internal diameter in systole; EF, ejection fraction; FS, fractional shortening. Data were mean ± SD.

### Metabolite assignment with NMR spectroscopy

**Figure 1:**
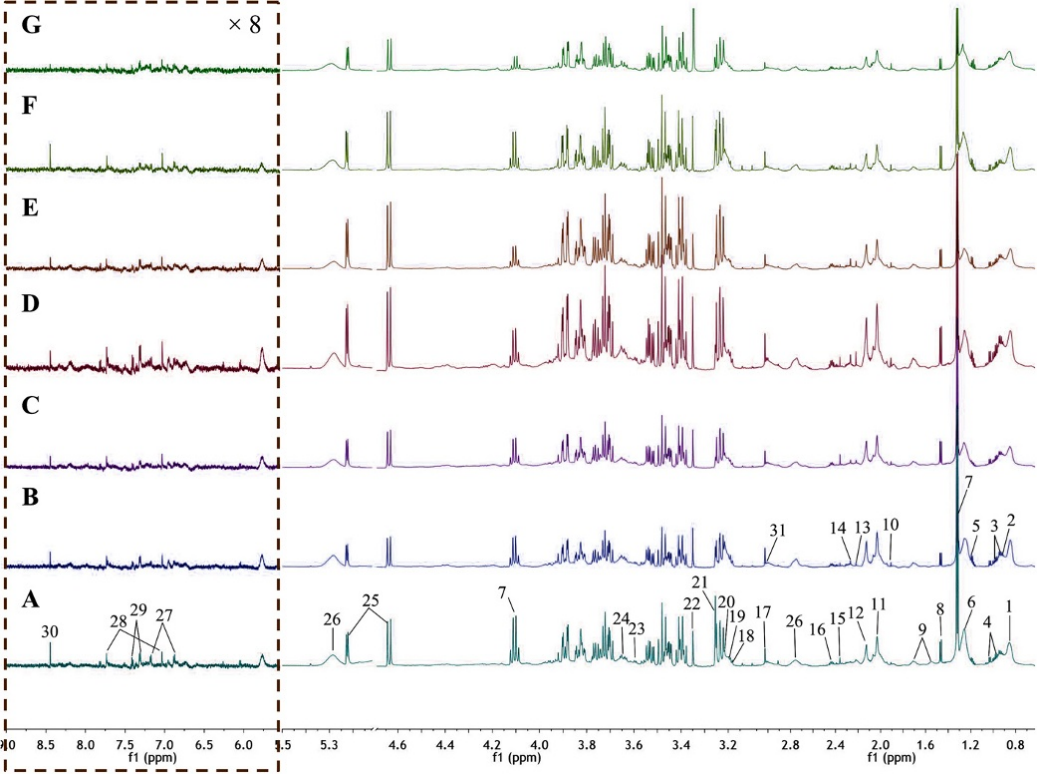
**Typical standard 1D** ^**1**^ **H NMR spectra of serum samples collected from rats in different groups. (A)** a sham rat; **(B)** a AMI rat; **(C)** a SGBO treated rat; **(D)** a SGB treated rat; **(E)** a ginsenosides-treated rat; **(F)** a ophiopogonin D-treated rat and **(G)** and a schizandrin-treated rat. All the spectra in aromatic region *δ* 5.6-9.0 were vertically expanded 8 times. The keys for metabolites were given in Table 2.

Typical ^1^H NMR spectra of serum samples collected on the 7th day from different groups were shown in Figure 1. The proton signals were assigned (Table 2) according to the literatures [15, 16] combined with in-house and public databases (http://www.hmdb.ca/). These assignments were further confirmed with COSY, TOCSY, *J*-res, HSQC, and HMBC 2D NMR spectra. Apart from the lipid signals generated from fatty acid (FA), triglyceride (TG), lipoproteins, and polyunsaturated lipids (PUFA), 26 metabolites were identified including glucose, glycerol, aliphatic amino acids (valine, isoleucine, leucine, alanine, glycine), acidic and basic amino acids (glutamate, lysine), aromatic amino acids (tyrosine, phenylalanine), amino acid derivatives (creatine, phosphocreatine, 1-methylhistidine), ketone bodies [*β*-hydroxybutyrate (*β*-HB), acetoacetate, acetone], carboxylicacids (acetate, formate), choline related metabolites [choline, phosphorylcholine (PC), glycerophosphocholine (GPC)], trimethylamine oxide (TMAO), glycolysismetabolite (lactate), *N*-acetyl and *O*-acetyl glycoproteins (NAG, OAG).

**Table 2:** ^**1**^ **H NMR data for metabolites in rat serum and significant changes of potential biomarkers**

No	Metabolites ^***a***^	Moieties	***δ*** ^1^H (multiplicity ^***b***^)	Selected signal	AMI/	GB/	OD/	SC/	SGBO/	SGB/
					Sham ^***c***^	AMI ^***c***^	AMI ^***c***^	AMI ^***c***^	AMI ^***c***^	AMI ^***c***^
1	lipoproteins	C*H*_3_(CH_2_)_n_ C*H*_3_CH_2_CH_2_C=	0.90 (br. t)		-	-	-	-	-	-
2	leucine	*δ*CH_3_, *δ′*CH_3_, *β*CH_2_, *γ*CH	1.00 (d), 1.02 (d), 1.67 (m), 1.73 (m), 3.75 (m)		-	-	-	-	-	-
3	isoleucine	*δ*CH_3_, *γ′*CH_3_, *γ*CH_2_, *β*CH, *α*CH	1.02 (t), 1.07 (d), 1.30 (m), 1.51 (m), 1.99 (m), 3.68 (d)		-	-	-	-	-	-
4	valine	*γ′*CH_3,_ *γ*CH_3,_ *β*CH, *α*CH	1.05 (d), 1.11 (d), 2.30 (m), 3.66 (d)		-	-	-	-	-	-
5	*β*-HB	CH_3_, CH_2_, CH	1.25 (d), 2.29 (m), 2.39 (m), 4.14 (m)	1.25	↓**	-	-	↑**	-	↑*
6	FA	CH_3_(C*H*_2_)_n_, C*H*_2_CH_2_CO, C*H*_2_-C = C, C*H*_2_-C = O, =C-C*H*_2_-C=	1.34 (m), 1.63 (m), 2.09 (m), 2.29 (m), 2.80 (m)	1.34	↑*	-	-	-	-	-
7	lactate	*β*CH_3_, *α*CH	1.39 (d), 4.18 (q)	1.39	↑***	-	-	-	↓***	↓**
8	alanine	*β*CH_3_, *α*CH	1.54 (d), 3.82 (q)		-	-	-	-	-	-
9	lysine	*γ*CH_2_, *δ*CH_2_, *β*CH_2_, *ϵ*CH_2_, αCH	1.53 (m), 1.75 (m), 1.94 (m), 3.05 (t), 3.83 (t)		-	-	-	-	-	-
10	acetate	CH_3_	1.98 (s)		-	-	-	-	-	-
11	NAG	CH_3_	2.09 (s)	2.09	↑*	-	-	-	-	-
12	OAG	CH_3_	2.19 (s)	2.19	↑***	-	-	-	↓**	↓**
13	acetone	CH_3_	2.28 (s)		-	-	-	-	-	-
14	acetoacetate	CH_3_, CH_2_	2.33 (s), 3.44 (s)		-	-	-	-	-	-
15	2-ketoglutarate	*β*CH_2_, *γ*CH_2_	2.46 (t), 3.03 (t)		-	-	-	-	-	-
16	glutamine	*β*CH_2_, *α*CH	2.19 (dd), 2.50 (dd), 3.83 (dd)		-	-	-	-	-	-
17	creatine/phosphocreatine	N-CH_3_, CH_2_	3.09 (s), 3.99 (s)	3.09	↑**	↓**	-	-	-	-
18	choline	N-(CH_3_)_3_, *α*CH_2_, *β*CH_2_	3.25 (s), 4.09 (t), 3.55 (t)	3.25	↓***	↑*	-	-	↑**	↑*
19	GPC	N-(CH_3_)_3_, *α*CH_2_, *β*CH_2_	3.26 (s), 4.32 (t), 3.69 (t)	3.26	↓***	↑*	-	-	-	↑**
20	PC	N-(CH_3_)_3_, *α*CH_2_, *β*CH_2_	3.27 (s), 4.16 (t), 3.60 (t)	3.27	↓**	↑*	-	-	-	↑*
21	TMAO	CH_3_	3.32(s)	3.32	↑**	-	-	-	-	-
22	methanol	CH_3_	3.42 (s)		-	-	-	-	-	-
23	glycine	CH_2_	3.61 (s)		-	-	-	-	-	-
24	glycerol	CH, CH_2_	3.81 (m), 3.71(m), 3.69 (m)	3.69	↑*	-	-	-	-	↓*
25	glucose	*α*C_1_H, *β*C_1_H, C_2_H	5.28 (d), 4.69 (d), 3.29 (dd)	5.28 4.69	↑***	↓**	-	-	↓*	↓**
26	PUFA	-CH = CH-, CH_2_	5.35 (m), 2.83 (m)	5.35	↑**	-	-	-	-	-
27	tyrosine	CH, CH	7.23 (m), 6.94 (m)	7.23	↑**	-	-	-	-	-
28	1-methylhistidine	C_4_H, C_2_H	7.09 (s), 7.81 (s)		-	-	-	-	-	-
29	phenylalanine	*α*CH, C_2_H/ C_6_H, C_4_H,C_3_H/ C_5_H	3.99 (m), 7.34 (m), 7.39 (m), 7.41 (m)		-	-	-	-	-	-
30	formate	*H*COOH	8.52 (s)	8.52	↑**	-	-	-	-	-
31	albumin	Lysyl-CH_2_	3.05 (m)		-	-	-	-	-	-

^*a*^Metabolites: LDL, low density lipoprotein; VLDL, very low density lipoprotein; *β*-HB, *β*-hydroxybutyrate; FA, fatty acid; NAG, *N*-acetyl-glycoprotein; OAG, *O*-acetyl-glycoprotein; GPC, glycerophosphocholine; PC, phosphocholine; TMAO, trimethylamineoxide; PUFA, polyunsaturated lipids.

^*b*^Multiplicity: singlet (s), doublets (d), triplets (t), doublet of doublets (dd), multiplets (m), quartets (q).

^*c*^Sh/C, G/C, D/C, S/C, SDG/C and SG/C represent sham group, schizandrin group, ophiopogonin D group, ginsenosides group, SDG group, and SG group compared to control group, respectively. ↑ indicates relative increase in signal, ↓ indicates relative decrease in signal, *represents a statistically significant difference (*p* < 0.05), **represents *p* < 0.01, ***represents *p* < 0.001.

Visual inspection of ^1^H NMR profiles revealed that the levels of some endogenous metabolites, such as lactate, glycoproteins, and glucose, changed evidently in Sham, SGB, and SGBO groups as compared with AMI model group. To reveal the effect of individual compound and combined treatment on metabolic profile modulation, we further performed multivariate analysis.

### Metabonomic changes in response to AMI in rats

**Figure 2:**
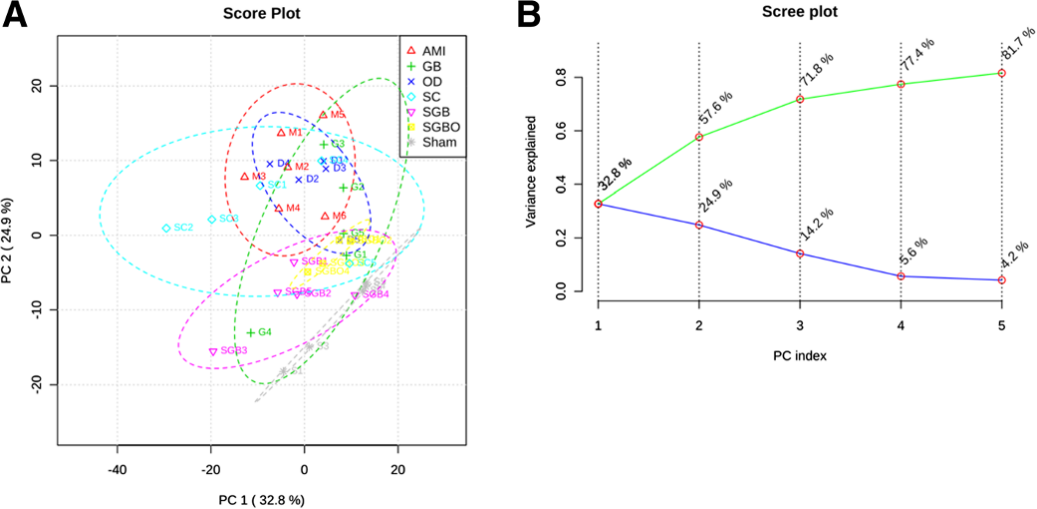
**Analysis results of PCA model.** The PCA score plot **(A)** and scree plot **(B)** of serum samples from 7 groups.

**Figure 3:**
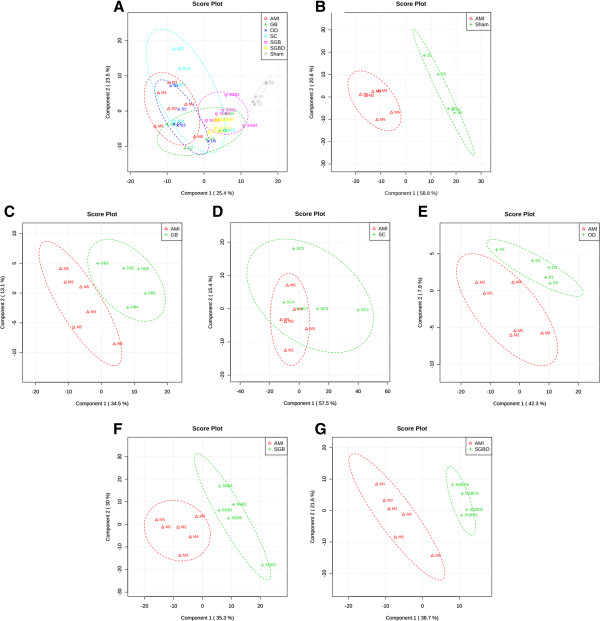
**Analysis results of PLS-DA model.** PLS-DA score plots of **(A)** 7 groups (R^2^ = 0.62, Q^2^ = 0.51); **(B)** AMI and sham groups (R^2^ = 0.91, Q^2^ = 0.83); **(C)** GB and AMI groups (R^2^ = 0.64, Q^2^ = 0.29); **(D)** SC and AMI groups (R^2^ = 0.22, Q^2^ = −0.15); **(E)** OD and AMI groups (R^2^ = 0.30, Q^2^ = −0.32); **(F)** SGB and AMI groups (R^2^ = 0.77, Q^2^ = 0.53); **(G)** SGBO and AMI groups (R^2^ = 0.82, Q^2^ = 0.60).

In this study, both PCA and PLS-DA approaches were used to distinguish between groups expected to show metabolic differences. The PCA score plot (Figure 2) exhibited that no outlier sample was detected within the NMR data (*δ* 0.5-4.34 and *δ* 6.24-9.0), and the ability of clustering was fair to distinguish the metabolic profile of rat in different groups. To obtain satisfactory classification and select candidate biomarkers, PLS-DA was further applied on two or more group data analysis (Figure 3A). Because of the poor signal to noise ratio, we re-analyzed the aromatic region (δ 6.24-9.0) separately, and expected to extract the differential information of aromatic amino acids. However, the clustering result of aromatic region from different samples was not acceptable on the score plot of PLS-DA, indicating the variables at the aromatic region had no contributing to group division.

**Figure 4:**
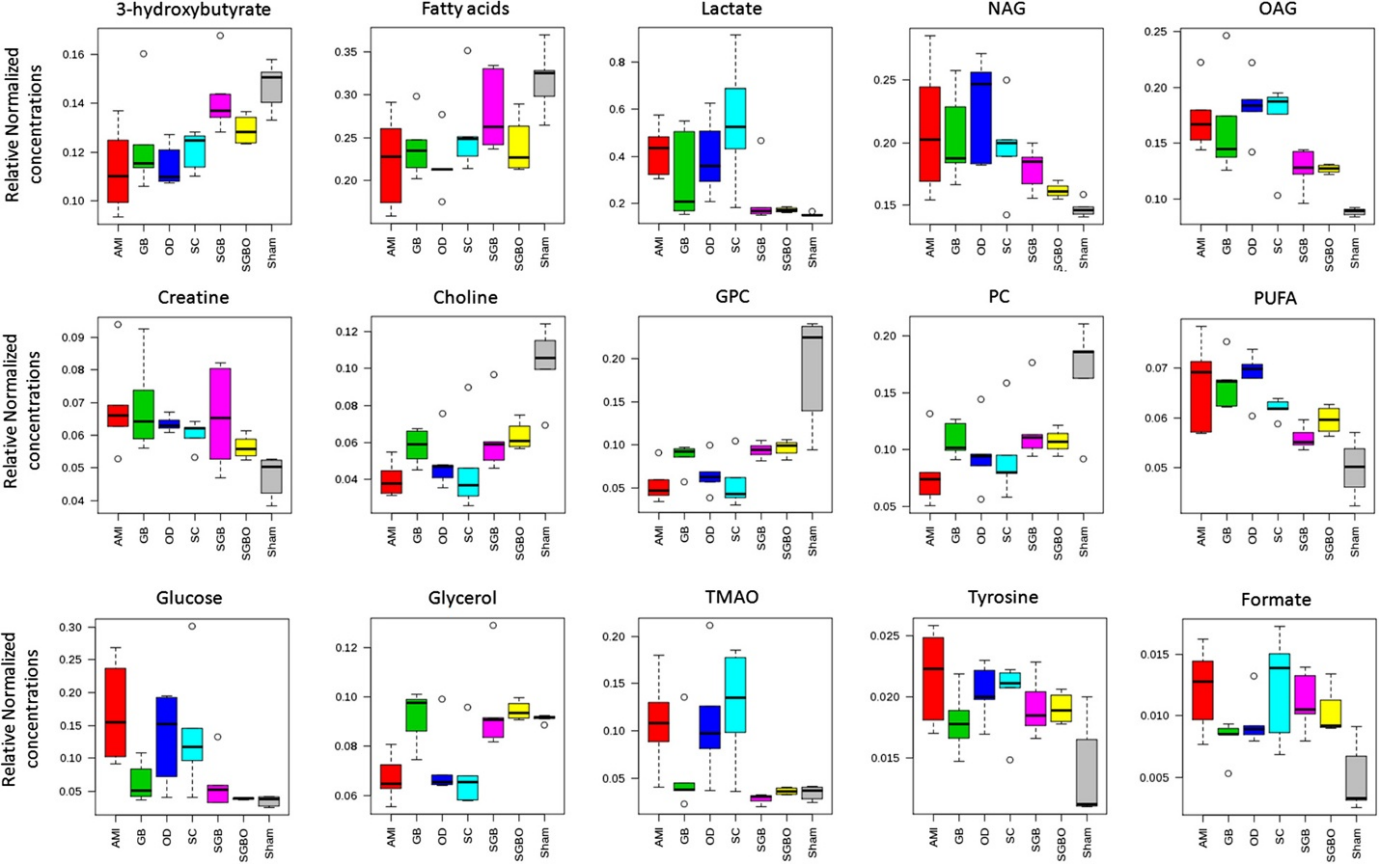
**Relative Normalized concentrations of the significantly changed metabolites.** Red, green, blue, light blue, pink, yellow and grey bar charts represent relative normalized concentrations in the AMI, GB, OD, SC, SGB, SGBO and sham group, respectively. NAG, N-acetyl-glycoprotein; OAG, O-acetyl-glycoprotein; GPC, glycerophosphocholine; PC, phosphocholine; TMAO, trimethylamineoxide; PUFA, polyunsaturated lipids.

There were three threshold used to select the metabolites that best correlate with the treatment options: (1) variables far from the origin point in the loading plots of PLS-DA (Additional file 1: Figure S1); (2) variables with VIP ≥ 1; (3) variables with statistical significant difference (*p* < 0.05, Additional file 2: Figure S2). The variables that satisfied the three thresholds at the same time could be chosen as potential markers. As shown in the PLS-DA score plot (Figure 3B), separation between sham and AMI groups was observed with an acceptable quality of fit and predictability (R^2^ = 0.91, Q^2^ = 0.83), indicating that significant metabolic changes were induced by AMI model. Compared with sham group, AMI models had significant elevation of 11 metabolites, including lactate, NAG, OAG, creatine, phosphocreatine, TMAO, glycerol, glucose, PUFA, tyrosine, and formate, together with decreased level for *β*-HB and choline-containing metabolites (Figure 4).

### Biological functions of potential biomarkers

**Figure 5:**
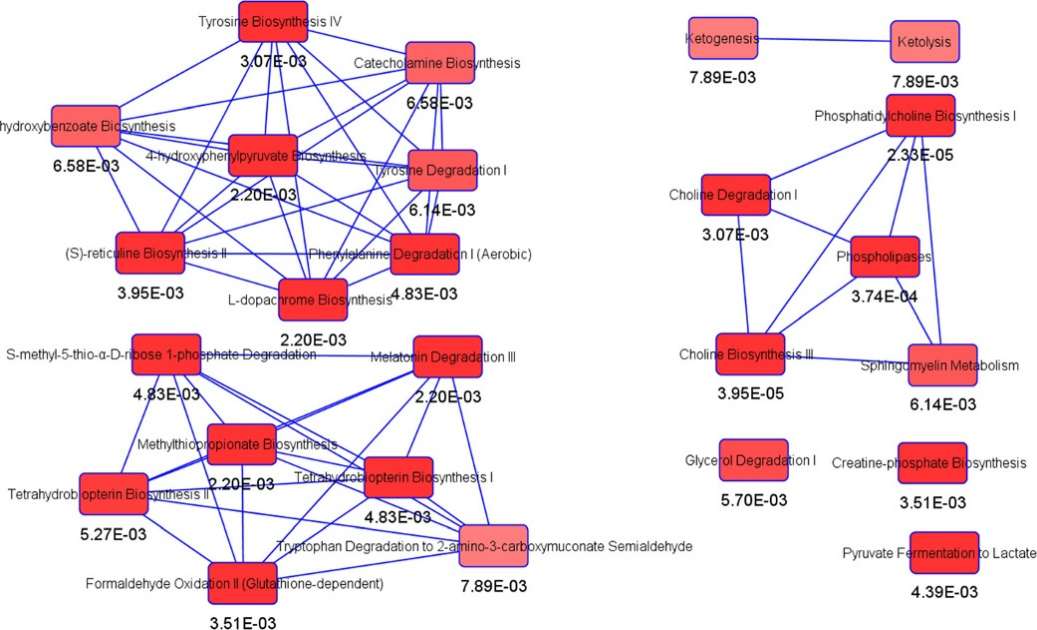
**Metabolic pathways of the differential metabolites.** Main canonical pathway with significance p < 0.05 related to the differential metabolites constructing by the Ingenuity Pathway Analysis (IPA).

A schematic diagram (Figure 5) was constructed according to the KEGG (http://www.genome.jp/kegg/) pathway database to investigate the relationships of the identified metabolites. These metabolites were mainly involved in fatty acid oxidation, glycolysis, arachidonic acid, linoleate, leukotriene, glycerophospholipid, phosphatidylinositol phosphate, and some amino acids metabolism.

The heart has a constant energy demand for adenosine triphosphate (ATP) to fulfill its continual contractile activity. Under normal physiological conditions, 90% of cardiac ATP is synthesized by mitochondrial oxidative phosphorylation of fatty acids [17]. Cardiac ischemia blocked the supply of oxygen and glucose in heart, leading to a switch in energy supply from aerobic metabolism to anaerobic metabolism [18]. Under hostile anaerobic conditions, glucose glycolysis becomes an important source to maintain ATP production [19]. As a result in the present study (Table 2), fatty acids were accumulated due to its decreased uptake and oxidation, and the level of glucose was increased from breakdown of stored glycogen in AMI group [20]. Accordingly, ketone body (e.g. *β*-HB) as the product of fatty acid oxidation was decreased [21], lactate as an end product of glucose anaerobic glycolysis was elevated in serum [22]. Moreover, an increased level of PUFA was observed in AMI rats, suggesting the inhibition of *β*-oxidation on unsaturated fatty acids [23]. Creatine is another important energy source abundant in both skeletal muscle and myocardial cells. With high energy demands, it is transported through blood and taken up by tissues. An increase of creatine concentration was exhibited in serum after AMI [24].

Cholines are essential elements of the cell membranes, and changes in their levels arerelated to cell membrane damages. Elevated plasma choline level has been proposed as an emerging biomarker associated with long-term risk of AMI but not a diagnostic marker of AMI at the time of emergency department presentation [25, 26]. In our case, the decreased levels of choline, PC and GPC were detected on the 7th day after AMI, which were opposite to the long-term observation. The stress response of early cell membrane damages may cause the down-regulation of the serum levels of cholines. TMAO is a gut microbe-dependent production of choline and synthesized in liver. The result of an increased level of TMAO in AMI rats was consistent with the previous report that TMAO was correlated with a risk of incident major adverse cardiovascular events [27]. An increased level of glycerol was detected in the model group, which indicated that myocardial infarction was possibly complicated with arrhythmias symptoms [28]. Acetylated glycoproteins, such as transferrin, α-1-antitrypsin or haptoglobin, are secreted by hepatocytes in response to tissue or cell membrane damage and act as inflammatory mediator [29–31]. The elevation in levels of NAG and OAG were likely to reflect an inflammatory response in AMI group.

### Regulatory effects of SGB and SGBO compared to mono-therapy

The PLS-DA score plot of 7 groups (Figure 3A) obviously showed that both SGB and SGBO treatments shifted the state from AMI model back towards sham group, while none of the mono-therapy group separated from AMI model significantly. Pairwise comparisons were carried out to find potential biomarkers responsible for the classification. The score plots between each treatment group and AMI model revealed that the combined treatment group of SGB (Figure 3F) and SGBO (Figure 3G) had significant differentiation with AMI group (R^2^ = 0.77, Q^2^ = 0.53; R^2^ = 0.82, Q^2^ = 0.60 respectively), whereas no difference was observed for mono-therapy groups of GB (Figure 3C), SC (Figure 3D), and OD (Figure 3E).

The details of selected potential biomarkers were summarized in Table 2 and Figure 5. In mono-therapy groups, *β*-HB was up-regulated by schizandrin, cholines and glucose were up-regulated by ginsenosides Rg1 and Rb1.It indicated that Schizandrin had a trend to improve fatty acid oxidation in AMI rats, and ginsenosides Rg1 and Rb1 might modulate the early stress response of cell membrane damages and promotes glycogenolysis and glycolysis to produce more ATP. Under the combined treatment, four biomarkers (lactate, choline, glucose, OAG) were significantly modulated toward the level of sham group in both SGB and SGBO treatment. A remarkable reduction of lactate suggested that improving myocardial energy metabolism was the major synergistic effect induced by combined-therapy. Four more biomarkers (*β*-HB, GPC, PC, glycerol) were regulated in SGB but not in SGBO treatment group, which indicated synergistic effects from combination therapy of SGB by regulating metabolic state. The concurrent level rise of *β*-HB and decrease of glucose further supported the notion of enhanced myocardial energy metabolism. The level increases for choline, PC and GPC and the decrease of glycerol were probably to overcome cell membrane damages. The level decrease for OAG was probably associated with the anti-inflammation activities of SGB and SGBO. These detailed mechanisms of synergy may provide insights on cardioprotection from a metabolic perspective.

## Conclusion

In the present study, the cardioprotective effect of the combination therapy with ginsenosides Rg1 and Rb1, Schizandrin, and Ophiopogonin D was evaluated on AMI rats using a metabonomic approach. Fifteen metabolites were significantly modulated after AMI, while 4 of them were modulated by SGBO treatment, 8 were modulated by SGB treatment, and only a few could be influenced by mono-therapy treatment. The results indicated the synergistic advantages of combined-therapy for preventing the endogenous metabolic disorders induced by AMI. Potential mechanisms were mainly related to improving the dysfunction of energy metabolism, as well as reconstructing cell membrane damages, and repairing inflammation injury. This study also demonstrated the potential of discovering new drugs through combinatorial bioactive components from traditional Chinese formula.

## Electronic supplementary material

Additional file 1: Figure S1: VIP scores and loading plots of PLS-DA. (A) between AMI and sham; (B) between GB and AMI; (C) between SC and AMI; (D) between OD and AMI; (E) between SGB and AMI; (F) between SGBO and AMI. (TIFF 3 MB)

Additional file 2: Figure S2: Heatmap visualization represented unsupervised hierarchical clustering. (A) between AMI and sham; (B) between SGB and AMI; (C) between SGBO and AMI. Rows: samples; Columns: top 50 NMR features ranked by t-test; Color key indicates feature expression value, blue: lowest, red: highest. (TIFF 2 MB)
