# Supplementary figures and images for: A metabonomic study of cardioprotection of ginsenosides, schizandrin, and ophiopogonin D against acute myocardial infarction in rats
Source: BMC Complement Altern Med. 2014 Sep 23;14:350. doi: 10.1186/1472-6882-14-350 (PMC4182767; doi:10.1186/1472-6882-14-350)

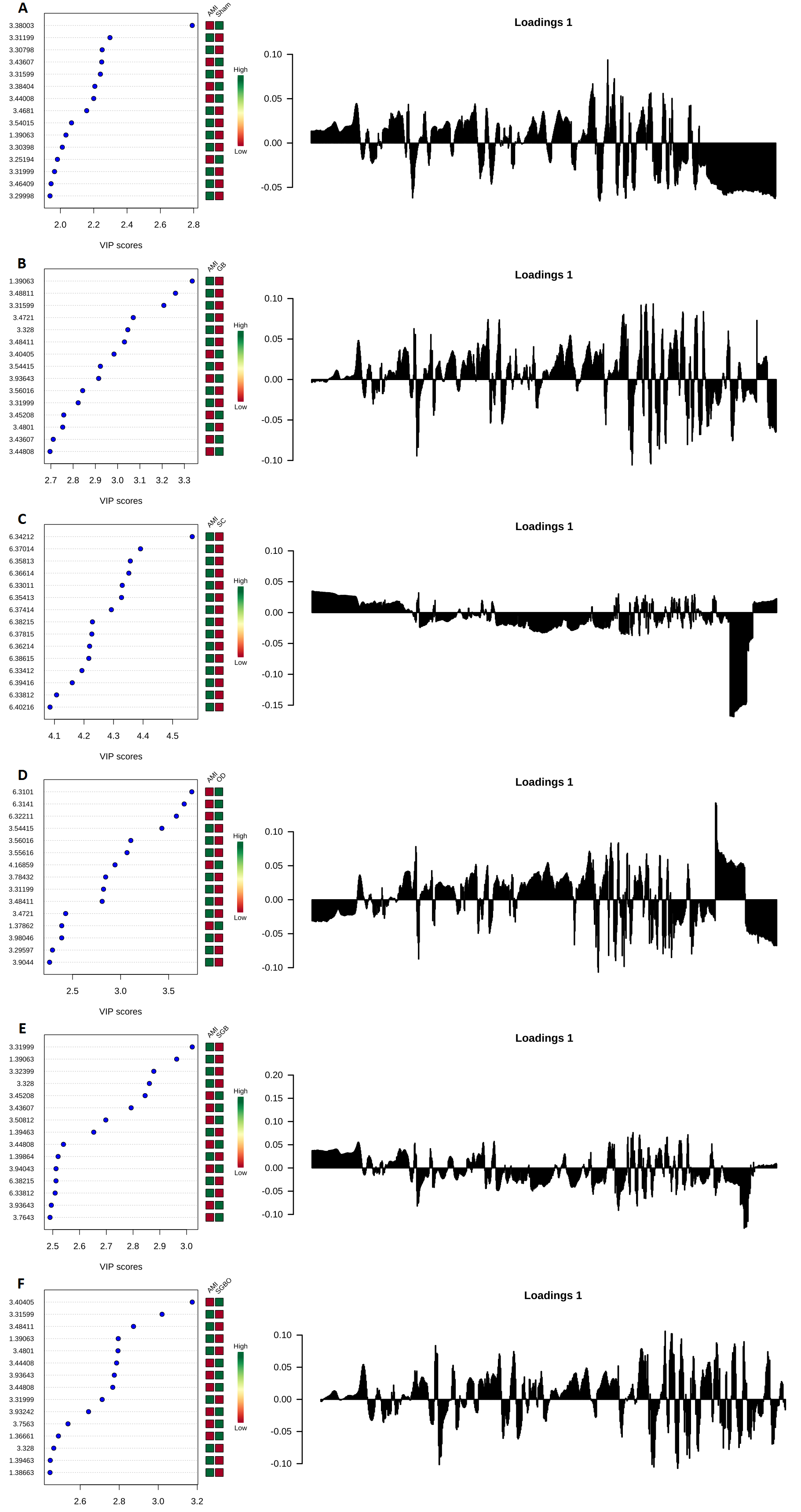

Supplement: Supplementary file 1 — Additional file 1: Figure S1: VIP scores and loading plots of PLS-DA. (A) between AMI and sham; (B) between GB and AMI; (C) between SC and AMI; (D) between OD and AMI; (E) between SGB and AMI; (F) between SGBO and AMI. (TIFF 3 MB) [file 12906_2014_1926_MOESM1_ESM.tiff]

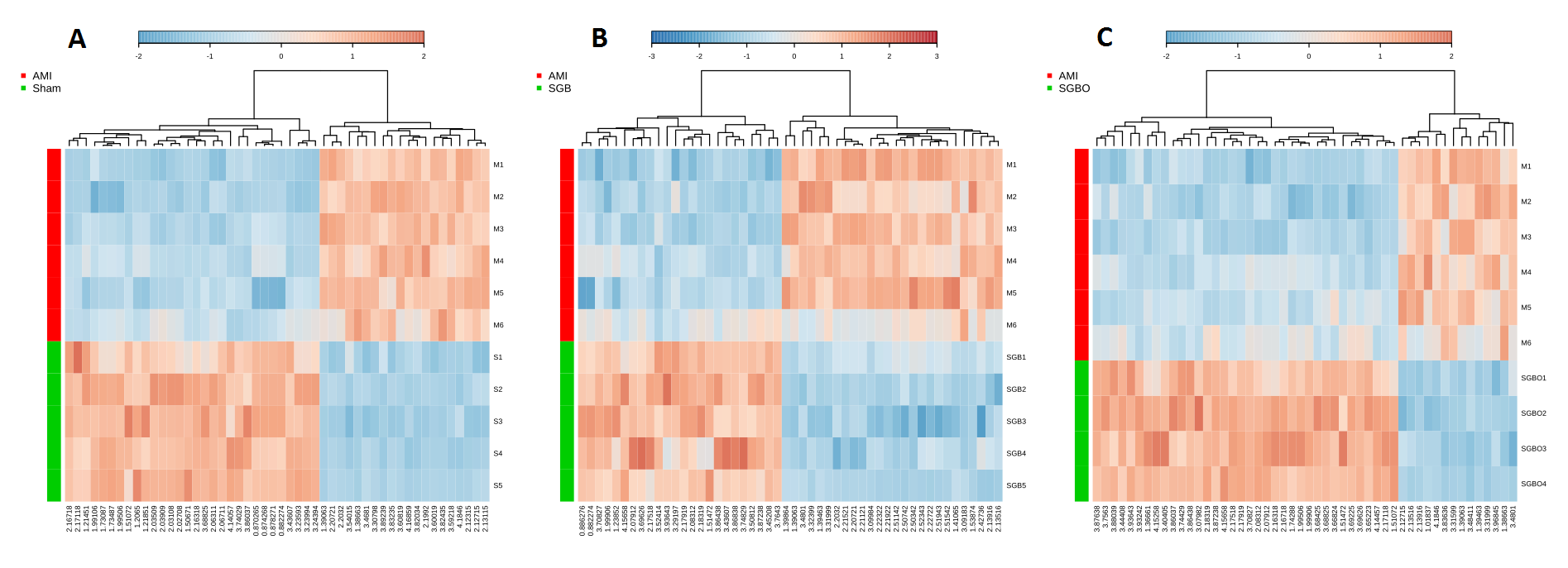

Supplement: Supplementary file 2 — Additional file 2: Figure S2: Heatmap visualization represented unsupervised hierarchical clustering. (A) between AMI and sham; (B) between SGB and AMI; (C) between SGBO and AMI. Rows: samples; Columns: top 50 NMR features ranked by t-test; Color key indicates feature expression value, blue: lowest, red: highest. (TIFF 2 MB) [file 12906_2014_1926_MOESM2_ESM.tiff]
